# Scrotal cancer: incidence, survival and second primary tumours in the Netherlands since 1989

**DOI:** 10.1038/sj.bjc.6605914

**Published:** 2010-09-28

**Authors:** R H A Verhoeven, W J Louwman, E L Koldewijn, T B J Demeyere, J W W Coebergh

**Affiliations:** 1 Department of Research, Eindhoven Cancer Registry, Comprehensive Cancer Centre South, PO Box 231, Eindhoven 5600 AE, The Netherlands; 2 Department of Urology, Catharina Hospital, PO Box 1350, Eindhoven 5602 ZA, The Netherlands; 3 PAMM Regional Laboratory for Pathology and Microbiology, PO Box 2, Veldhoven 5500 AA, The Netherlands; 4 Department of Public Health, Erasmus University Medical Centre Rotterdam, PO Box 2040, Rotterdam 3000 CA, The Netherlands

**Keywords:** scrotum, neoplasms, incidence, survival rate, neoplasms, second primary, carcinoma, squamous cell

## Abstract

**Background:**

Since the 1970s there have been few epidemiological studies of scrotal cancer. We report on the descriptive epidemiology of scrotal cancer in the Netherlands.

**Methods:**

Data on all scrotal cancer patients were obtained from the Netherlands Cancer Registry (NCR) in the period 1989–2006 and age-standardised incidence rates were calculated also according to histology and stage. Relative survival was calculated and multiple primary tumours were studied.

**Results:**

The overall incidence rate varied around 1.5 per 1 000 000 person-years, most frequently being squamous cell carcinoma (27%), basal cell carcinoma (19%) and Bowen's disease (15%). Overall 5-year relative survival was 82%, being 77% and 95% for patients with squamous and basal cell carcinoma, respectively. In all, 18% of the patients were diagnosed with a second primary tumour.

**Conclusion:**

The incidence rate of scrotal cancer did not decrease, although this was expected; affected patients might benefit from regular checkups for possible new cancers.

Pott (1775) described a relationship between soot exposure and a high incidence of scrotal cancer among chimney sweepers. Scrotal cancer has also been linked to exposure to tar (Henry, 1947; Waldron *et al*, 1984; Sorahan *et al*, 1989), pitch (Waldron *et al*, 1984; Sorahan *et al*, 1989), different types of lubricating and cutting oils (Kickham and Dufresne, 1967; Lee *et al*, 1972; Wahlberg, 1974; Lee, 1976; Roush *et al*, 1984; Waldron *et al*, 1984; Sorahan *et al*, 1989; Parys and Hutton, 1991; Coggon *et al*, 1996), creosotes (Heller, 1930; Henry, 1947), gas production (Henry, 1947; Doll *et al*, 1972), paraffin wax pressing (Lione and Denholm, 1959) and various treatments for skin diseases (Andrews *et al*, 1991; Stern and Laird, 1994; Stern *et al*, 2002). With the knowledge on occupational risk factors and the accompanying improvements in working conditions, scrotal has become rare. In the 1970s and early 1980s, its incidence in the United States was about one per million person-years, but has been increasing again since then (Roush *et al*, 1984; Weinstein *et al*, 1989; Goldoft and Weiss, 1992; Wright *et al*, 2008).

Scrotal cancer should not be confused with the more common testicular cancer, that mainly affects young adults. The scrotum is the protuberance of muscles and skin containing the testicles. Therefore most scrotal tumours are sarcomas or skin tumours whereas testicular cancers are usually germ-cell tumours. Tumours of the skin of the scrotum might also be classified as skin cancer, but owing to the historically strong aetiological relationship between scrotal tumours and occupational exposures and the lack of this relationship between occupational exposures and other tumours of the skin, scrotal tumours are classified as a separate entity.

Little information on the incidence and survival of scrotal cancer has been published in the last 20 years, probably because it was expected that the incidence would become almost zero after removal of the known scrotal carcinogens from the working environment. Given the relative lack of recent work, we have used recent Dutch data to investigate the stage distribution, histological distribution, incidence, survival and occurrence of second primary tumours in patients with this rare cancer since 1989.

## Materials and methods

Data were used from the nation-wide population-based Netherlands Cancer Registry (NCR), which combines the data from the eight Dutch regional cancer registries since 1989. These registries receive lists of newly diagnosed cancer patients on a regular basis from hospital pathology departments, all participating in a nation-wide network (PALGA). In addition, hospital medical records departments provide lists of diagnoses of outpatients and hospitalised cancer patients. Following these notifications, trained registrars extract patient and tumour characteristics (among other things, topography, histology, stage and date of diagnosis) data from the medical records. According to the registration rules of the NCR, scrotal skin tumours are categorised in the topographic group of scrotal cancer and not in the group of skin cancers.

Topography and histology were coded according to the International Classification of Diseases for Oncology (ICD-O; Fritz *et al*, 2000). All tumours with an ICD-O topography code scrotum (C63.2) were selected for this study. We grouped the histological codes according to the classification in Table 1. For all other analyses, lymphomas and mesotheliomas of the scrotum were excluded.

The stage of the squamous cell carcinomas, basal cell carcinomas, Paget's diseases and the tumours that were grouped in the histological ‘other’ group was categorised according to the IUCC carcinoma of the skin TNM classification (Sobin and Wittekind, 2002): stage 0 (TisN0M0), stage 1 (T1N0M0), stage 2 (T2–3N0M0), stage 3 (T4N0M0, any T N1M0) and stage 4 (any T, any N, M1). The stage of the melanomas diagnosed before 2003 was categorised according to the fifth TNM classification of malignant melanomas of the skin, and since 2003 and later, according to the sixth TNM classification (Sobin and Wittekind, 1997, 2002). Because Bowen's disease tumours are by definition *in situ* tumours and because sarcomas have no current TNM-classification these groups were excluded from the analyses according to stage.

Age and stage distributions were calculated according to histology. Five-year moving-average age-standardised incidence rates were calculated per 1 000 000 person-years for the entire group of scrotal cancers. Age-standardised incidence rates in 6-year diagnostic periods per 1 000 000 person-years were calculated according to histology. Standardisation of age was performed according to the European standard population. The Joinpoint regression program (v3.0, Statistical Research and Applications Branch, National Cancer Institute, Information Management Services, Inc., Silver Spring, MD, USA, http://www.srab.cancer.gov/joinpoint) was used to test whether there were increases or decreases in the overall incidence rate of scrotal cancer (Kim *et al*, 2000).

The frequency of patients with invasive primary tumours before and/or after scrotal cancer diagnosis was calculated. The strict rules of the NCR on registering second primary tumours ensured that only primary tumours were included in this analysis, as tumours with the same ICD-O topography group had to be diagnosed with a time difference of at least 6 months or had to belong to different morphological groups. For squamous cell carcinomas of the skin, only the first tumour could be included in the analyses owing to differences in registration methods over time and between regions.

Vital status data (available until 1 January 2008) were obtained from the hospital records and the mortality register of the Central Bureau for Genealogy (an institution that registers all deaths in the Netherlands via the municipal population registries). For patients with two scrotal tumours, only data on the first tumour were used for survival analyses. Relative survival was calculated for the total group and also according to histology. Relative survival is an estimation of the disease-specific survival, being the absolute survival among the scrotal cancer patients divided by the expected survival for the general population with the same sex and age structure (Hakulinen and Abeywickrama, 1985). Relative survival was computed by means of traditional cohort analysis.

## Results

**Figure 1:**
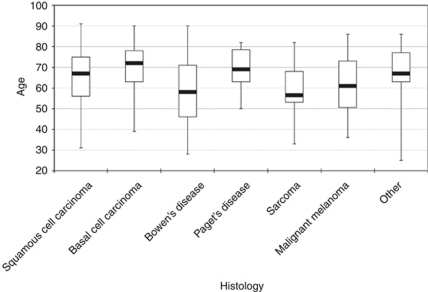
Age distribution of patients diagnosed with scrotal cancer in the Netherlands according to histology (median, Q1, Q3, lowest and highest age).

In all, 200 scrotal tumours in 194 patients were diagnosed in 1989–2006 in the Netherlands; their histology is shown in Table 1. The largest histological groups were squamous cell carcinomas (27%), basal cell carcinomas (19%), Bowen's disease (15%), sarcomas (13%) and extramammary Paget's disease (12%). Mesotheliomas and lymphomas were excluded from further analyses. Patients with basal cell carcinomas were oldest at diagnosis with a median age of 72 years, whereas patients with scrotal sarcoma had a median age of 56.5 years (Figure 1).

**Figure 2:**
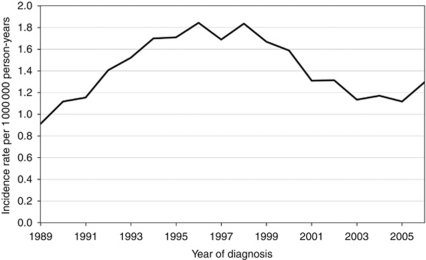
Five-year moving-average European standardised scrotal cancer incidence rates per 1 000 000 person-years (mesotheliomas and lymphomas excluded).

In the study period, the age-standardised 5-year moving-average incidence rate varied between 0.9 and 1.8 per 1 000 000 male person-years (Figure 2), with no statistically significant increase or decrease over time.

**Figure 3:**
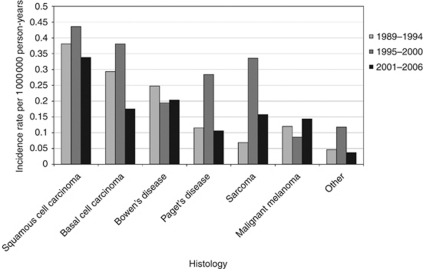
European standardised scrotal cancer incidence rates per 1 000 000 person-years according to histology.

Figure 3 presents age-standardised scrotal cancer incidence rates in 6-year periods according to histology. The highest incidence rates were found for squamous cell and basal cell carcinomas and the lowest incidence rates for the malignant melanomas and ‘other’ group. During 1995–2000, the incidence rates of scrotal squamous cell carcinoma, basal cell carcinoma, Paget's disease, sarcoma and the ‘other’ group seem to have increased temporarily. The stage distribution of the different histological groups is presented in Table 2. In all histological groups there are large percentages of tumours with an unknown stage, ranging from 13 to 47%. The majority of the patients with squamous cell carcinoma, basal cell carcinoma and Paget's disease had stage 1 or 2 tumours, whereas the majority of the malignant melanomas were stage 2 or 3.

Data on vital patients were missing for 34 (18%) of the 189 patients, most of them were diagnosed before 1995 (*n*=25). For this period follow-up of vital status is still incomplete in most of the regional cancer registries. Relative survival 1 year after diagnosis was 97% (95% confidence interval (95% CI): 91–100%), which decreased gradually to 82% (95% CI: 71–90%) 5-year relative survival and overall crude survival being 66% (95% CI: 58–74%). From 6 to 10 years after diagnosis, the relative survival remained about 80%, resulting in a 10-year relative survival of 77% (95% CI: 62–91%). Five-year survival estimates of patients with scrotal basal cell carcinomas, Bowen's diseases and sarcomas were 95% or higher, yet with wide CIs. The 5-year relative survival of patients with extramammary Paget's disease was 68% (95% CI: 36–94%), but 1- and 3-year relative survival estimates were both 100%. The 1-, 3- and 5-year relative survival of patients with squamous cell carcinomas was relatively low, being, respectively, 93% (95% CI: 79–100%), 80% (95% CI: 61–94%) and 77% (95% CI: 56–94%).

The distributions of the tumours diagnosed before or after the first diagnosis of scrotal cancer are presented in Table 3. A high percentage of skin tumours and tumours located near the scrotum (penis, prostate, anal canal, urinary bladder, colorectal, etc.) was found both before and after the diagnosis of scrotal cancer. Six men with a scrotal cancer (three with Bowen's disease, one with squamous cell carcinoma, one with basal cell carcinoma and one with some ‘other’ tumour) were diagnosed with a second scrotal tumour, three of these second scrotal tumours were squamous cell carcinomas, two belonged to the other category and one was a Bowen's disease. Four of these six patients also had other tumours (i.e. two cutaneous, one tumour of soft tissue, lung, larynx tumour or bladder, and one plasma cell tumour).

The 24 tumours detected before the diagnosis of scrotal cancer were found in 22 patients (12%) and 34 patients (18%) had at least one cancer diagnosis after the first scrotal cancer diagnosis in 1989–2006.

## Discussion

During 1989–2006, scrotal cancer occurred predominantly in men older than 50 years. The age-standardised incidence rate was around 1.5 per 1 000 000 person-years and did not seem to change over time. Squamous cell carcinomas were the most frequent histological type, followed by basal cell carcinomas. The stage of most tumours was unknown or low. The relative survival of scrotal patients was good. Multiple primary tumours were quite common among patients with scrotal cancer.

The incidence of scrotal cancer in the United Kingdom decreased during the 1970s and the early 1980s, probably because of previous improvement in the occupational hygiene and the removal of carcinogens; a further decrease in incidence was expected (Sorahan *et al*, 1989). However, although we found a relatively steady incidence, a recent American study found an increase since the early 1980s (Wright *et al*, 2008). This may indicate that not only occupational exposures influence the risk of scrotal cancer, which is also indicated by the histological distribution. In this study, 27% of the tumours were squamous cell carcinomas and 15% Bowen's disease (*in situ* squamous cell carcinomas), whereas previous studies reported that the great majority were squamous cell carcinomas (up to 93% Kickham and Dufresne, 1967; Ray and Whitmore, 1977; McDonald, 1982; Parys and Hutton, 1991). The recent US study reported a similar percentage of squamous cell carcinomas (32%) as our study (Wright *et al*, 2008). Because almost all occupationally caused scrotal tumours were squamous cell carcinomas (Kickham and Dufresne, 1967), the current low proportion of such tumours may indicate that certain non-occupational exposures are relevant. Possible non-occupational risk factors are sun exposure, several types of treatments for skin diseases and the human papilloma virus (Andrews *et al*, 1991; Stern and Laird, 1994; Guran and Pak, 1999; Stern *et al*, 2002; de Vries *et al*, 2006).

The relatively high percentage of tumours with unknown stage in this study probably reflects the very good prognosis of most tumours. In general, these superficial tumours are surgically removed without further staging or treatment. If most tumours with unknown stage tumours are indeed of lower stage, the percentage of lower stage (stage 0, 1 or 2) would be around 90%, being somewhat higher than that in other studies (McDonald, 1982; Roush *et al*, 1985; Wright *et al*, 2008).

Both our study and the recently published study in the United States found that survival of patients with squamous cell carcinomas of the scrotum seems to be lower than that of patients with a scrotal basal cell carcinoma or sarcoma (Wright *et al*, 2008). Some extra therapeutic caution may thus be needed for scrotal squamous cell carcinomas. The 5-year relative survival of scrotal squamous cell carcinoma patients (77%) was also lower, although not significantly, than the 5-year relative survival of male skin squamous cell carcinoma patients in the Netherlands (91%) (http://www.ikcnet.nl/page.php?id=224). Previous studies have not calculated relative survival based on reasonable numbers of scrotal cancer patients. A study in a region of the United Kingdom including 324 patients diagnosed in 1936–1976 reported crude 5-year survival estimates of 51%, with no change over time (Waldron *et al*, 1984), which, albeit being significantly lower than our crude 5-year survival of 66%, is not largely different.

Of scrotal cancer patients, 18% developed one or more tumours after the scrotal tumour and six (3%) patients developed a second scrotal cancer. All of these tumours were diagnosed in the period 1989–2006, so patients who were diagnosed recently only had a short time to manifest a second (scrotal) tumour, and a longer follow-up would probably reveal a higher percentage of second (scrotal) cancers. This also applies to tumours that were diagnosed before the scrotal cancer diagnosis. However, the number of scrotal tumours and the number of tumours diagnosed after a first scrotal tumour could also have been increased by increased surveillance on new tumours by medical specialists in the patients who were already diagnosed with a previous tumour. Regular follow-up of scrotum cancer patients might thus be useful to detect new tumours at an early stage, but might also result in overdiagnosis.

In another study, 8 of the 19 patients with a squamous cell carcinoma of the scrotum had a cancer in their medical history, 5 of these 8 previous cancers were skin cancers (Weinstein *et al*, 1989). A patient series from the UK reported 69 (20%) of the 344 scrotal cancer patients to have second primary tumours, similar to our study (Waldron *et al*, 1984). It is generally known that people with skin tumours have a high chance of developing more skin cancers; scrotal skin cancer does not seem to be an exception (Marcil and Stern, 2000).

We found the incidence of scrotal cancer to be relatively stable, varying between 0.9 and 1.8 per million person-years, although we had expected a decreasing trend. The largest histological groups were the squamous cell carcinomas, basal cell carcinomas and Bowen's disease. The 5-year relative survival for the whole group of scrotal cancer patients was high (81%). The high percentage of second primary tumours after scrotal cancers suggests that scrotal cancer patients might benefit from regular checkups for possible new cancers.

## Figures and Tables

**Table 1:** Histological classification and distribution of scrotal cancer patients in the Netherlands

**Histological group**	**Morphology code according to ICD-O (Fritz *et al*, 2000)**	**No.**	**%**
Squamous cell carcinoma and variants	8033, 8070, 8071, 8072, 8076, 8083, 8094	53	27
Basal cell carcinoma	8090, 8091, 8096	38	19
Bowen's disease	8081	29	15
Sarcoma	8804, 8810, 8850, 8851, 8852, 8853, 8857, 8890, 8900	26	13
Extramammary Paget's disease	8542	24	12
Malignant melanoma	8720, 8721, 8743	16	8
Lymphoma	9675, 9680, 9699	4	2
Mesothelioma	9050	1	1
Other	8000, 8010, 8051, 8140, 8247, 8400, 8402, 8410, 8830	9	5
Total		200	100

Abbreviation: ICD-O=International Classification of Diseases for Oncology.

**Table 2:** Number of scrotal tumours according to stage and histology

**Stage**	**Squamous cell carcinoma**	**Basal cell carcinoma**	**Paget's disease**	**Malignant melanoma**	**Other**
0	1	0	2	0	1
1	22	12	2	1	2
2	18	8	9	6	1
3	2	0	2	6	1
4	0	0	0	1	1
Unknown	10	18	9	2	3
Total	53	38	24	16	9

The histological groups ‘sarcomas’, ‘lymphomas’ and ‘mesotheliomas’ do not have a TNM stage distribution and the ‘Bowen's disease’ are by definition *in situ* tumours; therefore these histologies are not included in this table.

**Table 3:** Tumours of scrotal cancer patients diagnosed before and after the first diagnosis of scrotal cancer

**Site**	**Number of tumours before scrotal cancer**	**Number of tumours after scrotal cancer**
Skin, squamous cell carcinoma	9	7
Lung	1	8
Colorectal	5	4
Scrotum	NA	6
Prostate	2	4
Non-Hodgkin's lymphoma	2	1
Urinary bladder	2	1
Anal canal	1	1
Skin, melanoma	1	1
Chronic myeloproliferative disorder	1	0
Eye	0	1
Larynx	0	1
Pancreas	0	1
Penis	0	1
Plasma cell tumour	0	1
Primary site unknown	0	1
Skin, other	0	1
Soft tissue	0	1
Total	24	41

Abbreviation: NA=not applicable.
